# *In-vivo* longitudinal imaging of microvascular changes in irradiated oral mucosa of radiotherapy cancer patients using optical coherence tomography

**DOI:** 10.1038/s41598-017-16823-2

**Published:** 2017-11-28

**Authors:** A. V. Maslennikova, M. A. Sirotkina, A. A. Moiseev, E. S. Finagina, S. Y. Ksenofontov, G. V. Gelikonov, L. A. Matveev, E. B. Kiseleva, V. Y. Zaitsev, E. V. Zagaynova, F. I. Feldchtein, N. D. Gladkova, A. Vitkin

**Affiliations:** 1 Nizhny Novgorod State Medical Academy, Minina Square 10/1, 603005 Nizhny Novgorod, Russia; 2 Lobachevsky University, Gagarin Ave 23, 603950 Nizhny Novgorod, Russia; 3 Institute of Applied Physics Russian Academy of Sciences, Ulyanova Street 46, 603950 Nizhny Novgorod, Russia; 4 University of Toronto and University Health Network, 610 University Ave., Toronto, Ontario, M5G 2M9 Canada

## Abstract

Mucositis is the limiting toxicity of radio(chemo)therapy of head and neck cancer. Diagnostics, prophylaxis and correction of this condition demand new accurate and objective approaches. Here we report on an *in vivo* longitudinal monitoring of the oral mucosa dynamics in 25 patients during the course of radiotherapy of oropharyngeal and nasopharyngeal cancer using multifunctional optical coherence tomography (OCT). A spectral domain OCT system with a specially-designed oral imaging probe was used. Microvasculature visualization was based on temporal speckle variations of the full complex signal evaluated by high-pass filtering of 3D data along the slow scan axis. Angiographic image quantification demonstrated an increase of the vascular density and total length of capillary-like-vessels before visual signs or clinical symptoms of mucositis occur. Especially significant microvascular changes compared to their initial levels occurred when grade two and three mucositis developed. Further, microvascular reaction was seen to be dose-level dependent. OCT monitoring in radiotherapy offers a non-invasive, convenient, label-free quantifiable structural and functional volumetric imaging method suitable for longitudinal human patient studies, furnishing fundamental radiobiological insights and potentially providing useful feedback data to enable adaptive radiotherapy (ART).

## Introduction

The painful inflammation and possible ulceration of the mucous membranes lining the digestive tract known as mucositis is most common limiting toxicity of radio(chemo)therapy of head and neck cancer^1,2^. Its pathogenesis is complex and is based on the interaction of various cell and tissue factors, including both the effects of the oral cavity microflora and the development of vascular reactions^3–5^. Activation of the coagulation system is also known to play an important role in the development of acute radiation reactions^6^. The direct effect of the exposure of mast and endothelial cells to ionizing radiation is the generation of thrombin, and the release of histamine and prostaglandins I2 and Е2, leading to vasodilation and to increased vascular permeability resulting from the adhesion of neutrophils to the endothelial surface in the first several hours following irradiation^7–10^.

All modern systems of grading the severity of radiation mucositis are based on patient symptoms and complaints, and visual changes in the mucosae identified during examination of the oral cavity; these cannot provide objective and quantifiable information^11^. Experimental studies using repeated biopsies and histological investigations for evaluating reactions of the microstructure of the mucosa have been reported^12^, but these are rare and have little appeal for clinical practice adoption. Clearly, a noninvasive *in-vivo* method for detecting subclinical and quantifiable changes of the irradiated mucosa that are impossible to register during a standard visual examination is highly desirable. It would enable currently-impossible clinical tasks, including (1) detecting mucosal reactions before the onset of their clinical manifestations, thus offering the possibility of early intervention in suitable patients, (2) properly evaluating the effectiveness of different preventive measures and treatments of the mucositis, (3) monitoring of the radiobiological response of the different mucosal components such as small versus large vessels RT effects, their temporal dynamics relative to mucositis manifestation and relative to structural tissue changes (e.g., epithelium nature and thickness), and (4) possible alterations in the radiation treatment delivery in the context of ART (adaptive radiotherapy^13^). Such a method should be ideally noninvasive, contrast-agent-free, suitable for repeated *in-vivo* investigations, well tolerated by patients, and provide useful information in real-time; the ability to detect tissue functionality including mucosal microcirculation during the course and after irradiation would also be desirable^14,15^.

Optical coherence tomography (OCT) is a medical noninvasive imaging modality that seems suitable for the task mentioned above at hand, with some pre-clinical and clinical experience in the diagnosis of malignant tumors and detailed *in-vivo* tissue assessment for over 20 years^16,17^. In recent years, works have been published on OCT application in evaluating the consequences of radiation/chemoradiation therapy of oral/pharyngeal tumors^18,19^. It has been previously shown that normal oral mucosa is easily accessible for OCT imaging with a suitably-designed probe, and exhibits a well-delineated high-contrast stratified structure on OCT^19^.

In addition to high-resolution microstructural tissue imaging, alternate OCT contrast mechanisms have enabled sensitive imaging of the microvasculature based on Doppler- and speckle-based OCT^20,21^.

Recent microvascular OCT studies in the oral mucosa of healthy human volunteers have also been performed^22^. Experimental preclinical^23^ and preliminary clinical studies^18^ have examined the possibility of OCT monitoring of the onset and progression of radiation mucositis (but without microvascular assessment). Wilder-Smith *et al*.^24^ offered a semi-quantitative method based on the combined evaluation of structural OCT changes and damage to the microcirculation in chemotherapy-induced oral mucositis on hamsters as determined by Doppler studies^24^ (although unable to detect/assess the fine changes in capillary-like vessels studied here). OCT studies for monitoring *late* oral radiation toxicity based on microstructural and limited microvascular data (including approximate categorizing of vessels with diameter >50 μm) have also been reported^25,26^. Given this promising research activity, the potential importance of blood microcirculation in mucositis pathogenesis^27^, and recent technological advances in multifunctional OCT (including microcirculation imaging^28^, with its much improved microvascular visualization and quantification capabilities), a pilot study of microstructural and microvascular OCT *in-vivo* imaging in head and neck radiotherapy patients is reported. Our OCT study quantifies RT-induced changes in the microvasculature, including in smallest capillary-like (<15 μm in diameter) vessels in the human oral mucosa of radiotherapy patients. Fine blood microcirculation details can potentially be detected by other techniques such as direct oral microscopy^29^, conventional capillary microscopy^30,31^, orthogonal polarization spectroscopy (OPS)^32^, side stream dark field (SDF) imaging^31–34^, and narrow band imaging (NBI)^35–38^. In comparison to these, OCT looks particularly promising due to its signal information content, imaging speed, resolution, penetration depth, access to different clinical sites via flexible fiber optic probes, and relative robustness in realistic clinical imaging conditions (e.g., tissue/patient motion artefacts).

To the best of our knowledge, previous works do not closely overlap with the current study and its intent to (1) show the technical feasibility of OCT monitoring in radiotherapy patients (with focus on quantifying changes in capillary-like vasculature), (2) evaluate its compatibility with clinical radiotherapy patient workflow, and (3) elucidate the initial radiobiological trends of mucosal tissue response (including mucositis development).

## Results

**Figure 1:**
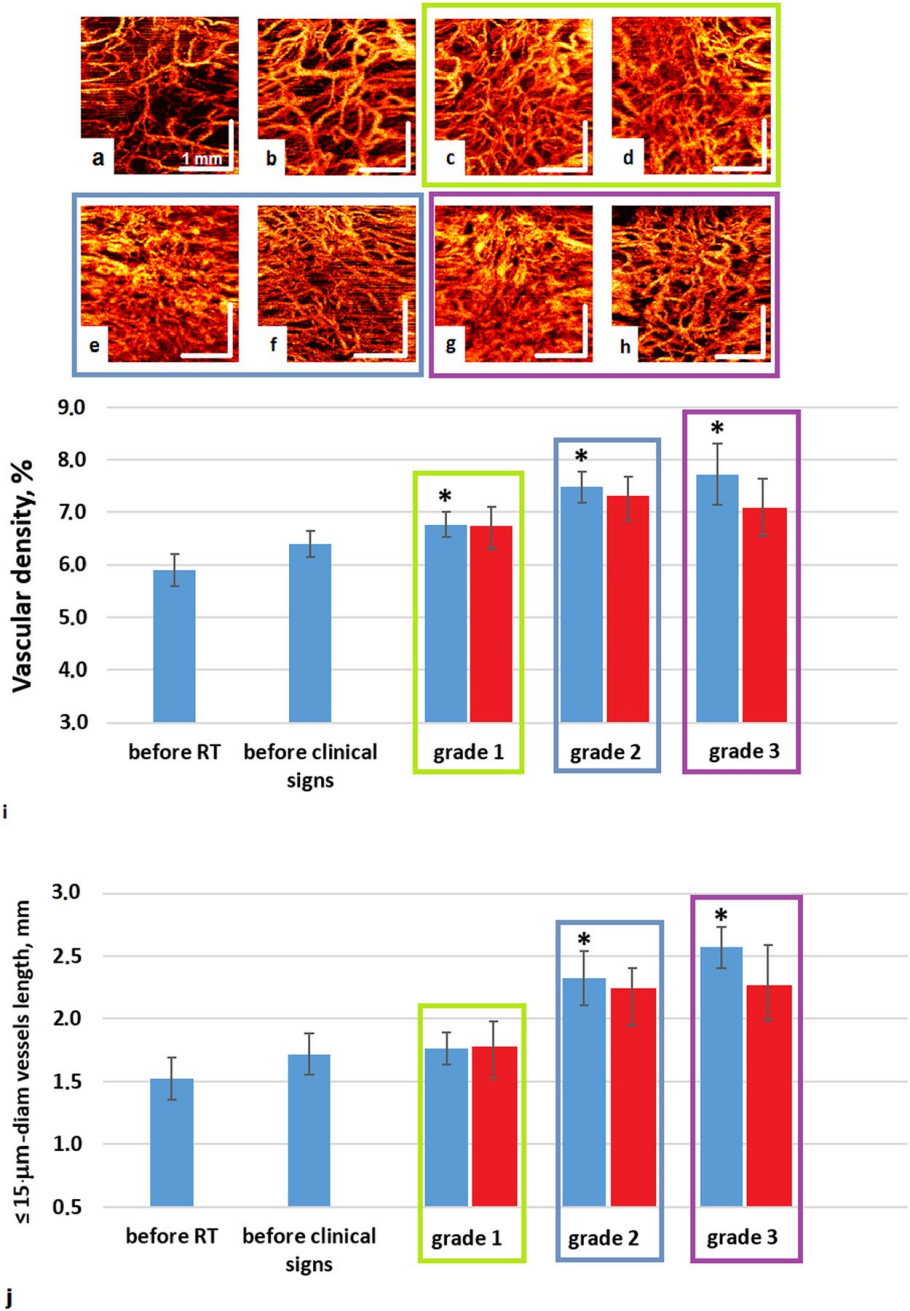
MIP OCT angiography for monitoring oral mucosal reaction to radiation. Representative svOCT images (**a**) – before RT; (**b**) - before visual signs of mucositis appear (after 8 Gy), an increase of vascular density is observed; (**c**) – grade 1 mucositis (10–12 Gy); (d) – after initiation of anti-mucositis therapy; (**e**) - grade 2 mucositis (after 14 Gy); (**f**) – after initiation of anti-mucositis therapy; (**g**) - grade 3 mucositis (after 20 Gy); (**h**) - after initiation of anti-mucositis therapy. Corresponding to these 8 representative svOCT image panels, summary statistics from the entire patient cohort are summarized in (**i**) – average vascular density; and (**j**) – total length of <15-μm-diameter vessels. In (**i**,**j**), data shown are mean ± SD; number of analyzed 3D image data sets and number of patients = 61 and 25 (before RT), 57 and 25 (before visual signs), 19 and 5 (grade 1 mucositis), 21 and 5 (after initiation of anti-mucositis therapy), 20 and 12 (grade 2 mucositis), 24 and 12 (after initiation of anti-mucositis therapy), 21 and 5 (grade 3 mucositis), 22 and 5 (after initiation of anti-mucositis therapy). Blue bar is a peak of symptoms of mucositis; red bar is after initiation of anti-mucositis therapy (see text for details). *Statistically significant difference compared to pre-RT levels (one-tailed t-test, p ≤ 0.05).

The OCT angiographic images of the normal mucosa show dense, volumetrically uniform microvascular networks, mostly consisting of relatively large vessels (Fig. 1a). At doses of 4–8 Gy, all patients exhibited an increased vessels density (Fig. 1b). Continued dose accumulation caused an increase in the clinical manifestations of radiation reaction, as also seen by the 2D OCT angiographic maps (Fig. 1c,e,g). After start of anti-mucositis therapy (typically chamomile and antiseptic washes in case of grade 1 (Fig. 1d); washes and analgesics in case of grade 2 (Fig. 1f); analgesics, antibacterial and antifungal therapy in case of grade 3 (Fig. 1h)), only in the case of grade 3 mucositis, the anti-mucositis therapy tended to slow down the microvascular increase (Fig. 1h). These tentative visual conclusions were confirmed by quantitative processing of the OCT images; we chose two quantification metrics to analyze these 2D OCT angiographs, as described in Materials and Methods section (Fig. 1i,j). Especially significant changes of microvascular parameters (compared to their pre-RT values) were detected when grade two and three mucositis developed (Fig. 1i,j).

**Figure 2:**
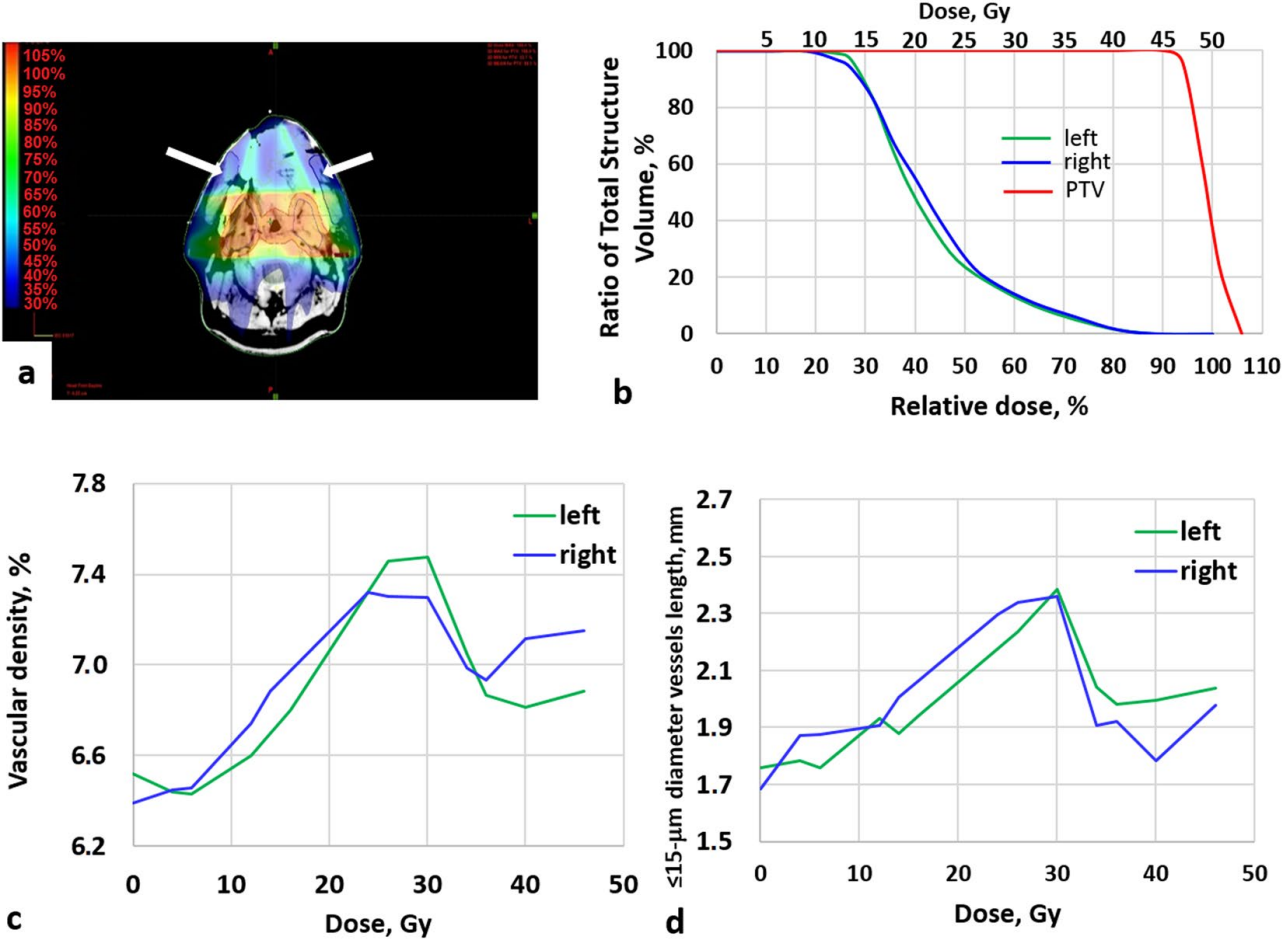
Changes of oral mucosa microvasculature during IMRT (*Case report 1*). (**a**) – Dose distribution, with dose heat maps (hotter colours = higher dose), with arrows indicating the OCT imaging locations; (**b**) – DVH of PTV and oral mucosa of both cheeks; (**c**) – vascular density; (**d**) – total length of <15-μm-diameter vessels. The first clinical symptoms of mucositis occurred at 22 Gy, after which the patient was treated by chamomile and antiseptic washes. Note that both microvascular metrics exhibit significant changes much earlier than the clinical manifestation of mucositis on both cheeks.

**Figure 3:**
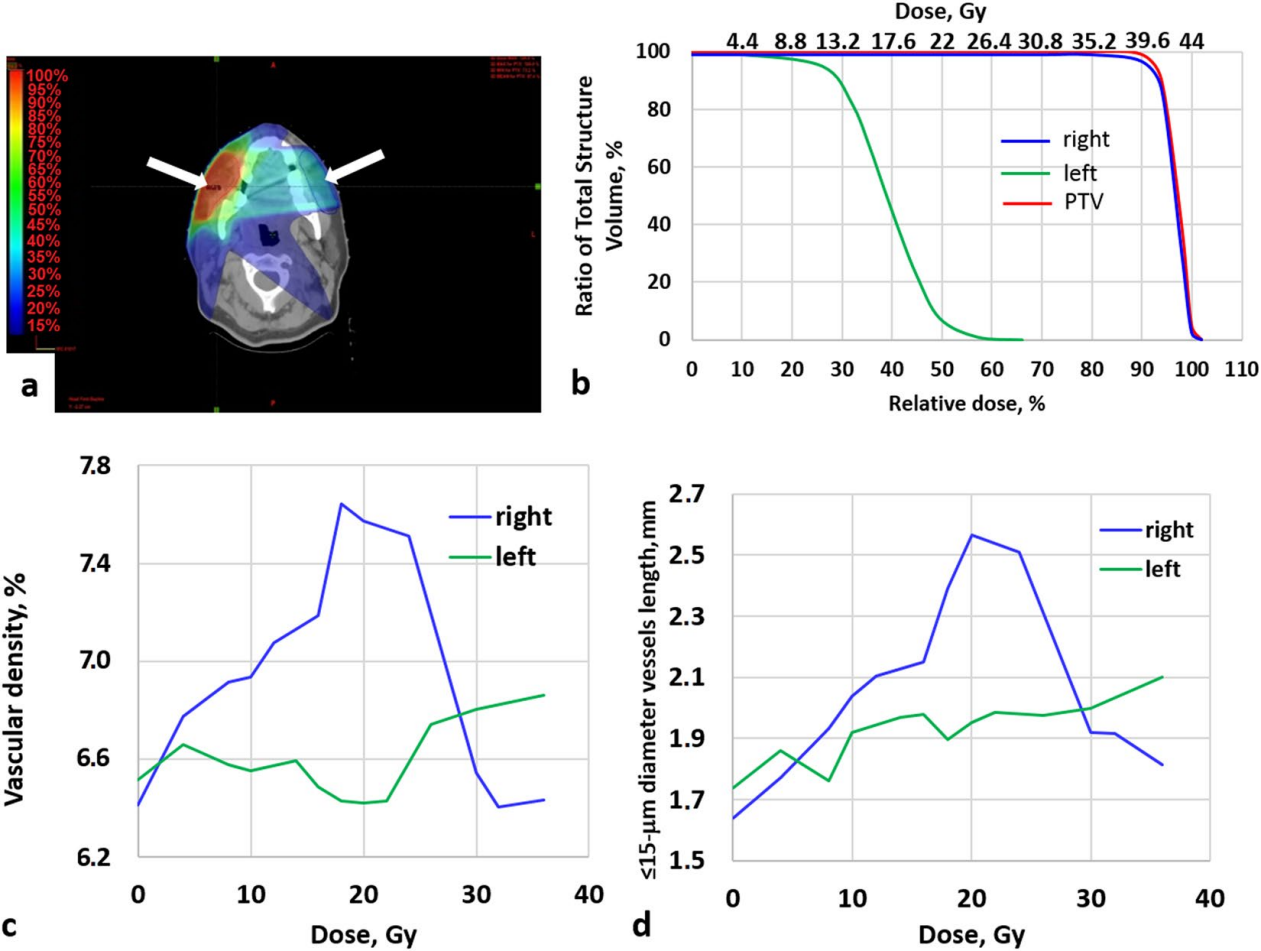
Changes of microvasculature of oral mucosa throughout the IMRT course (*Case report 2*). (**a**) – Dose distribution, with dose heat maps (hotter colors = higher dose, with arrows indicating the OCT imaging locations; (**b**) – DVH of PTV and oral mucosa of both cheeks: (**c**) – vascular density; (**d**) – total length of < 15-μm-diameter vessels. The patient was treated by chamomile and antiseptic washes to combat RT toxicity (after ~16 Gy on the right cheek; left cheek mucositis manifestations were very limited; for details, see text). Note that both microvascular metrics exhibit significant changes much earlier than the clinical manifestation of mucositis on the right cheek.

In addition to these general trends based on the analysis of all examined patients, the real potential of OCT microvascular monitoring was evident in patients who received Intensity Modulated Radiation Therapy (IMRT). The first clinical symptoms of mucositis usually occurred after accumulation of 10–15 Gy in the buccal tissue. This may correspond to different dose accumulated in the Planning Target Volume (PTV), dependent on PTV location and treatment plan. Two case reports are presented below (Figs. 2 and 3).

*Case report 1*: male, age 55. Diagnosis: nasopharyngeal cancer, T2N2M0, a low-differentiated squamous cell cancer. Before radiation therapy, four cycles of inductive chemotherapy were carried out. Patient was treated by IMRT and, accordingly, the treatment plan involved a conformal dose distribution with high dose gradients near the normal tissue borders. The Dose-Volume Histogram (DVH) for right and left cheek (monitoring sites) were similar (Fig. 2b) and corresponded to 40–45% of tumor (PTV) dose levels (Fig. 2a). The first clinical symptoms of mucosal reaction (hyperemia) occurred after a total PTV dose of 22 Gy, corresponding to ~10 Gy on right and left buccal mucosa.

Mucositis reached no more than first degree for 10 days. After beginning of radiation reaction (occurring at the total PTV dose of ~22 Gy), the patient was treated by chamomile and antiseptic washes. After that, visual symptoms of mucositis began decreasing.

OCT monitoring of the buccal mucosa showed similar microvascular changes in the right and left cheeks (Fig. 2c,d), and the appearance of a vascular reaction prior to clinical manifestations and symptoms of mucositis. At their maxima at doses of ~30 Gy, the total length of <15-μm-diameter vessels increased by 40% and 35% on the right and left cheeks, respectively. The similarity in the values the microvascular metrics and their time-course changes for the two cheeks is indicative of their similar doses; importantly, these metrics show significant changes well before clinical manifestations of mucositis.

*Case report* 2: female, age 34. Diagnosis: cancer of the minor salivary gland of the right cheek T1N0M0, mucoepidermoid carcinoma after a non-radical resection of the tumor. Postoperative IMRT (Fig. 3a) on the tumor bed was performed with total dose = 44 Gy, dose/fraction = 2 Gy (22 fractions). In this patient, the PTV and right cheek’s tissue including mucosa coincided. The first clinical symptoms of oral mucosa reaction (hyperemia) occurred after a total PTV dose of ~14 Gy. Since the left cheek was more distant from PTV region, its dose was smaller, which caused asymmetry in the right/left cheek responses. The appearance of second grade mucositis (patches and erosions) occurred after a dose of 20 Gy on the right cheek and persisted for 6 days. On the left cheek mucosa, the severity of mucositis was less significant and was limited to hyperemia and single fibrous patch.

Analysis of OCT images of the right cheek indicated the increase of the total length of <15-μm-diameter vessels and the vascular density immediately after beginning of radiation therapy (Fig. 3c,d). When the total dose was 8 Gy, the number of small vessels on the right buccal mucosa increased by 17.5%, and at the peak of the reaction (dose ~20–22 Gy) by more than 56%. After beginning of antibacterial (anti-mucositis) therapy, these indicators decreased and reached an initial level at the dose of 30 Gy. We also detected and quantified the difference in the microvascular changes on the right and left cheeks, as expected from their different dosimetry (Fig. 3b). The number of <15-μm-diameter vessels on the left buccal mucosa showed a much smaller increase, reaching its maximum of 21% at a PTV dose of 36 Gy that corresponded to a dose of approximately 10 Gy to left cheek mucosal tissue.

## Discussion

The development and clinical adoption of conformal irradiation including IMRT improved dose delivery to the tumor and decreased dose received by organs at risk. However, the significant problem of oral cavity mucositis has not disappeared. In fact, it seems as if IMRT’s highly conformal dose distributions and high dose gradients in both pathologic and normal tissues have made its appearance and severity more unpredictable and plan/patient specific^39^. Hence, diagnosis, prophylaxis and corrective treatments of radiation induced mucosal toxicity demand correspondingly more accurate and objective approaches. OCT is a non-invasive, label- and contact-free, convenient, high-resolution 3D structural and functional subsurface imaging modality that may meet some of these demands, including early detection prior to clinical manifestation of toxicities. While other technologies may potentially be adapted to monitor microvascular changes in RT patients (e.g., direct oral microscopy^29^, conventional capillary microscopy^30,31^, orthogonal polarization spectroscopy^32^, side stream dark field imaging^31–34^, narrow band imaging^35–38^, laser Doppler flowmetry, high-frequency Doppler ultrasound, and confocal or intravital endoscopy^40^), OCT’s unique combination of capabilities and strengths bodes well for its increased use in many potential scenarios in radiotherapy, in particular those with microvascular involvement.

This study reports the first use of OCT for *in-vivo* longitudinal monitoring of microvascular monitoring of oral mucosa in the course of radio(chemo)therapy in patients with oral, oropharyngeal and nasopharyngeal cancer. Microvascular reactions as imaged and quantified by OCT were dose-level dependent and were detected *before* clinical mucositis was observed. These findings may have relevance in the context of early intervention in patients who, on the basis of early-stage OCT microvascular data, are likely to develop severe oral complications; as such, this may enable data-informed adoption of ART scenarios. In the cases of IMRT patients, similarity or dis-similarity of microvascular metrics in the different mucosal sites tracked with corresponding doses/DVHs (Figs. 2 and 3). The total dose to the mucosa of each cheek depended on the treatment plan and in some cases (e.g., IMRT) could significantly differ from the right and left sides. In this and similar situations, OCT proved to be a very useful tool to identify subtle dose-level-dependent changes in the microcirculation, which are not determined by visual inspection alone.

Further, the study demonstrated the technical feasibility and clinical use of a prototype OCT system suitable for *in-vivo*, longitudinal, objective and quantifiable high-resolution imaging of the subsurface microstructure and microvasculature of oral mucosa in RT patients. Importantly, the OCT examination procedure was well tolerated by most consenting patients.

The presented OCT vascular imaging and quantification method has some methodological limitations. Indeed, mucositis is a complex pathological process that affects not only tissue microvasculature but its microstructure/morphology as well (e.g., thickness of mucosal layers, scattering properties). As these in turn may (slightly) affect OCT vessel visualization, the derived microvascular metrics may also reflect slight contributions from tissue morphology alterations.

Further, motion artefacts are typically present in OCT angiographic images and reduce their quality. Attempts to eliminate/compensate for the artefacts may lead to loss of the useful signal as well (like in other *in vivo* imaging techniques). Further development of robust algorithms to avoid these losses are needed.

Overall, this study represents early stage phase I research with a limited patient cohort, and more work is needed to better assess technological feasibility and potential clinical utility of OCT microangiography in radiotherapy. In particular, more extensive exploration of correlation between the early OCT-detected mucosal microvascular changes in the course of RT and the early and late RT toxicities is planned. Further research will also encompass a more thorough mucosal assessment by multifunctional OCT, including quantitative evaluation of the microstructure of the mucous membrane layers and the state of its connective tissue matrix (via polarization-sensitive OCT). This will provide more accurate information about subtle changes in the various mucosal compartments (epithelium, connective tissue, microcirculatory bed) during irradiation. Objective testing of the efficacy of various agents for the prevention and treatment of mucositis via multifunctional OCT is also envisioned.

## Conclusion

The study showed that longitudinal OCT angiographic monitoring can be used for objective evaluation of radiation induced microvascular volumetric changes in the oral mucosa, thus ‘shedding light’ on the temporal sequence of early functional and structural radiation toxicities. This may potentially play a role in the design and effectiveness evaluation of anti-mucositis treatment and prophylaxis modalities, and in the implementation of adaptive radiotherapy protocols.

## Materials and Methods

### Patients’ characteristics

Longitudinal imaging results from twenty-five (25) patients with stage I–IV of oral, oropharyngeal and nasopharyngeal squamous cell carcinoma are reported. Patient study was performed in the department of radiation oncology of Nizhny Novgorod Regional Oncology hospital and approved by the Research Ethics Board of the Nizhny Novgorod State Medical Academy. Informed consent was obtained from all participants and/or their legal guardians enrolled in the study. All methods were performed in accordance with the relevant guidelines and regulations. The patient characteristics and treatment methods are summarized in Table 1. All target volumes were irradiated to a total dose of 46–70 Gy as deemed clinically useful for tumor cure, but which can also cause radiation side-effects in the mucosa (oral cavity radio-toxicity such as mucositis). Irradiation was performed using a linear accelerator (Varian Clinac 600) or Cobalt-60 system (Terabalt). Mucositis degree was scored by Radiation Therapy Oncology Group and European Organization for Research and Treatment of Cancer (RTOG/EORTC) scale. All 25 patients received prophylaxis and treatment of mucositis according to our hospital standards (diet and oral care). From the first day of RT, patients were advised to rinse oral cavity and pharynx by chamomile as often as possible. Patients with grade 2 mucositis (single erosions and plaques) were assigned antiseptic washes and analgesics. For grade 3 mucositis, analgesics, antibacterial and antifungal therapy were assigned.

**Table 1:** Patient and radiotherapy characteristics for the examined cohort (n = 25).

**Gender**	**Men**			**Women**	
	23			2	
**Age**	38–64				
**Tumor location**	Oral cavity			Oropharynx	Nasopharynx
	tongue	bottom of the mouth	alveolar ridge		
	8	3	3	8	3
**Tumor stage**	I	II	III	IV	
	1	8	9	7	
**Therapy modalities**					
Radio(chemo)therapy (curative intent), total dose = 66–70 Gy, 2 Gy fractions		Preoperative radiotherapy, total dose = 46 Gy, 2 Gy fractions		Postoperative radiotherapy, total dose = 50 Gy, 2 Gy fractions	
21		3		1	
**Treatment plan**	5 = IMRT; 20 = 3D conformal				
Severity of developed mucositis	3 = grade 0; 5 = grade 1; 12 = grade 2; 5 = grade 3				

### Multifunctional OCT imaging

**Figure 4:**
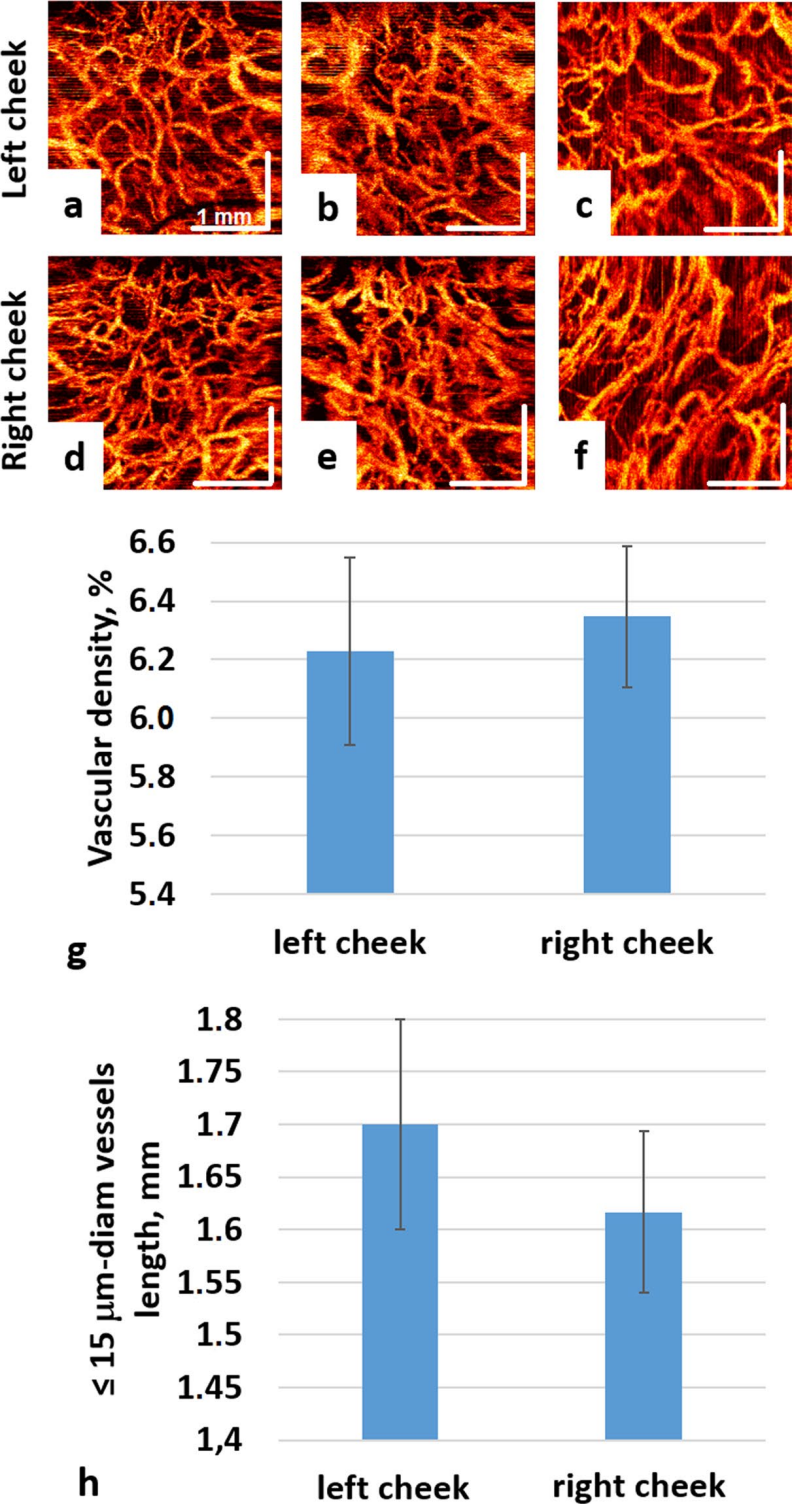
Robustness/reproducibility of OCT angiography monitoring at ~ same anatomical sites on the left and right cheeks obtained at three separate measurements (see text for details). (**g**,**h**) - quantification of OCT angiographic images: (**g**) - vascular density; (**h**) − ≤15 mm-diam vessels length. Data presentes as mean ± SD. No statistically significant differences were found between separate measurements near same anatomical reference points.

OCT imaging was performed twice a week, starting from the first day of irradiation or chemotherapy on two standard, symmetric sites inside both cheeks (Fig. 4). In all cases, the minimal distance from visual tumor (PTV) border to the cheek monitoring sites was >3 cm, which allowed us to minimize the influence of tumor angiogenesis effects. A modified ophthalmic head restrainer was used, with chin rest and forehead strap ensuring adequate immobilization for the OCT imaging session. After initial exploration and mapping of the oral cavity, sites along the centre line connecting the secretory duct of the salivary gland and the angle of the mouth were chosen. Such anatomical referencing allows for reproducible OCT angiographic images (Fig. 4).

Buccal mucosa (cheek tissue) was selected for OCT monitoring. It is an accessible, convenient and clinically important site for the introduction and reliable positioning of the OCT probe, which is important for image quality; this location also minimizes patient discomfort during the imaging study. It was verified that the OCT images of the buccal mucosa were visually and qualitatively similar with the probe re-positioning of ±several millimeters; therefore, probe positioning based on visual landmarks was sufficient to provide consistent results. Typically, six 3D data sets from two sites in each patient (with three repeats) were collected during each imaging session. In the pre-RT examinations, no statistically significant differences were found between left and right cheek within three repeats as shown in Fig. 4(g,h) (although the tumor was asymmetrically located at the left side of the tongue in this case). This demonstrates our ability for robust probe repositioning in the context of a longitudianl imaging study. Overall ~70% of OCT angiographic images were deemed visually appropriate, but for accurate quantification of microvascular parameters only ~40% were used, since some of these actually suffered motion artefacts manifest as bright stripes on OCT angiographic images. The OCT imaging procedures were well tolerated by consenting patients.

### OCT setup

**Figure 5:**
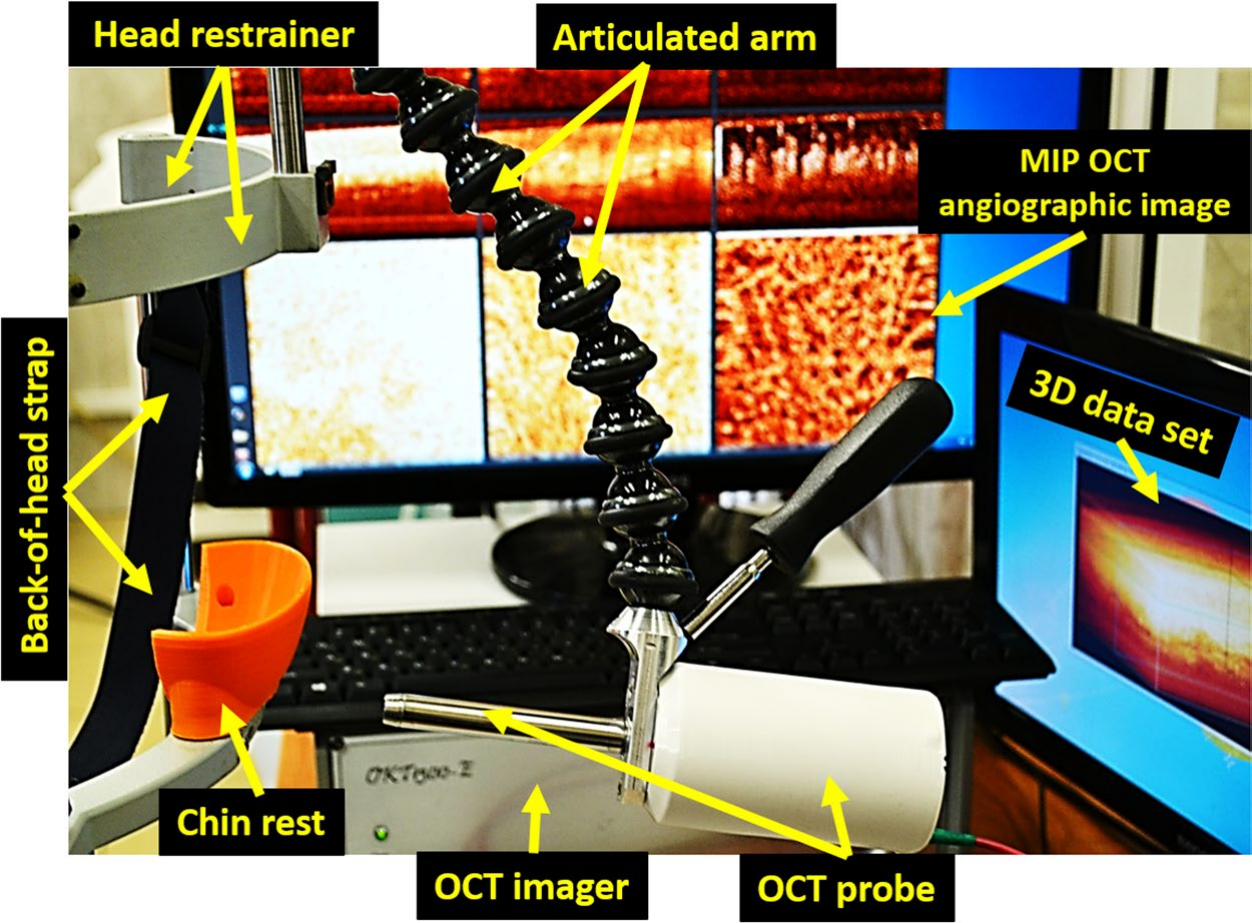
OCT setup for patient monitoring, with real-time images displayed in the background.

A spectral domain OCT (SD OCT) system operating at 1.3 μm central wavelength with axial resolution of ~10 μm and lateral resolution of ~15 μm in air with an imaging speed of 20,000 A-scans/sec was built, drawing on our previous experience^41–43^. Infrared laser power incident on tissue was low (~2 mW), and the imaging sessions were relatively fast (26 seconds beam-on scanning time, ~3 minutes total for two oral mucosa locations including set-up and alignment). The oral OCT probe (7 cm length × 1.1 cm diameter) was based on common-path interferometry scheme to provide convenient operation during the clinical study. The electromechanically actuated cantilever with optical fiber tip and focusing micro-lens was placed in the stainless steel tube with fused silica output window, to provide biocompatibility and ease of sterilization between imaging sessions. Patient’s head was immobilized in a head support frame to minimize motion artefacts. The optical probe was positioned at the oral mucosa site with gentle contact using an articulated arm apparatus (Fig. 5). The lens system formed a 15 µm dimeter focal spot 500 µm distal to the end face of the probe’s output window.

Based on temporal speckle variations as the source of angiographic image contrast, 3D OCT angiographic images (3 mm × 3 mm laterally, ~1.5 mm depth) were obtained. The angiographic calculation algorithm was based on image phase alignment to reduce motion artefacts and high-pass filtering along slow scanning axis^28,44^. Optically linearized spectrometer based on our previous development^40^ reduced signal computational complexity and allowed real time visualization of angiographic images. Between patients, the probe underwent cold chemical sterilization in a polyhexamethylene biguanide hydrochloride solution.

### Processing of OCT images

For OCT angiographic analysis and quantification, each obtained 3D microvascular network was converted to 2D images with Maximum Intensity Projection (MIP) by projecting the whole imaged depth (~1 mm) onto the plane. The choice of 2D analysis helps to minimize the influence of shadow artefact cast by flowing blood below the true vessels^45^. Resulting 2D images were binarized and skeletonized. Vessel thickness was calculated as 2x the distance between the vessel’s binary image border and its skeleton; vessels with overlapped binary borders and skeletons were assigned a thickness of 1-pixel. For the lateral resolution of the current OCT imager, this corresponds to vessel diameters less than ~15 μm. The total length of such thinnest vessels served as one quantified microvascular metric. The second derived metric was vessel density, which was calculated as the number of pixels of all vessel skeletons in the analyzed image area divided by the total number of pixels in this area.

Other more advanced microvascular quantification biomarkers are currently being explored (fractal dimension, Euclidian segment lengths, etc – see for example^46^), but these two selected ones sufficed for the current feasibility study.

### Statistical analysis

Statistical analysis used the one-tailed Student’s t-test (Statistica 6.0), with p ≤ 0.05 being considered statistically significant. The values of the two reported microvascular metrics throughout the course of therapy were compared to pre-RT levels.

### Data availability statement

The datasets generated during and/or analysed during the current study are available from the corresponding author on reasonable request.

## References

[1] Dörr W (2010). Radiation effect in normal tissue – principles of damage and protection. Nuklearmedizin.

[2] Sonis ST (2004). The pathobiology of mucositis. Nature Reviews Cancer.

[3] Scully C, Epstein J, Sonis S (2003). Oral mucositis: A challenging complication of radiotherapy, chemotherapy, and radiochemotherapy: Part 1, pathogenesis and prophylaxis of mucositis. Head & Neck.

[4] Sonis ST (2009). Mucositis: The impact, biology and therapeutic opportunities of oral mucositis. Oral Oncology.

[5] Sonis ST (2000). Defining mechanisms of action of interleukin-11 on the progression of radiation-induced oral mucositis in hamsters. Oral Oncology.

[6] Denham JW, Hauer-Jensen M (2002). The radiotherapeutic injury – a complex ‘wound’. Radiotherapy & Oncology.

[7] Molla M (1999). Influence of dose-rate on inflammatory damage and adhesion molecule expression after abdominal radiation in the rat. International Journal of Radiation Oncology, Biology, Physics.

[8] Panes J, Anderson DC, Miyasaka M, Granger DN (1995). Role of leukocyte-endothelial cell adhesion in radiation-induced microvascular dysfunction in rats. Gastroenterology.

[9] Dunn MM, Drab EA, Rubin DB (1986). Effects of irradiation on endothelial cell-polymorphonuclear leukocyte interactions. Journal of Applied Physiology.

[10] Molla M, Panes J (2007). Radiation-induced intestinal inflammation. World Journal of Gastroenterology.

[11] Cancer Therapy Evaluation Program, Common Terminology Criteria for Adverse Events, version 3.0, DCTC, NCI, NIH, DNNS. Available from: http://ctep.cancer.gov (2009).

[12] Richter C, Jaal J, Kuschel M, Doerr W (2007). Recombinant human keratinocyte growth factor modulates inflammatory changes in mouse oral mucosa during fractionated irradiation. International Journal of Radiation Oncology Biology, Physics.

[13] Schwartz DL (2013). Adaptive radiotherapy for head and neck cancer-dosimetric results from a prospective clinical trial. Radiotherapy and Oncology: Journal of European Society for Therapeutic Radiology and Oncology.

[14] Fajardo, L.F., Berthrong, M., Anderson, R.E. *Radiation pathology* 454 (New York: Oxford University Press, Inc., 2001).

[15] Baker DG, Krochak RJ (1989). The response of the microvascular system to radiation: a review. Cancer Invest.

[16] Schmitt JM (1999). Optical coherence tomography (OCT): a review. IEEE Journal of Selected Topics in Quantum Electronics.

[17] Adie, S.G., Boppart, S.A. Optical coherence tomography for cancer detection in *Optical Imaging of**Cancer. Clinical Application* (ed. Rosenthal, E., Zinn, K.R.) 209–250 (Springer, 2009).

[18] Gladkova N (2008). Application of optical coherence tomography in the diagnosis of mucositis in patients with head and neck cancer during a course of radio(chemo)therapy. Medical Laser Application.

[19] Gladkova, N. D. *et al*. Optical coherence tomography in dentistry. *Handbook of Biophotonics*. *Photonics for Health Care* (ed. Popp, J., Tuchin, V., Chiou, A., Heinemann, S. H.) 2, 1029–1040 (Wiley-VCH, 2012).

[20] Standish BA (2008). Interstitial Doppler optical coherence tomography as a local tumor necrosis predictor in photodynamic therapy of prostatic carcinoma: an *in vivo* study. Cancer Research.

[21] Mariampillai A (2010). Optimized speckle variance OCT imaging of microvasculature. Optics Letters.

[22] Choi WJ, Wang RK (2014). *In vivo* imaging of functional microvasculature within tissue beds of oral and nasal cavities by swept-source optical coherence tomography with a forward/side-viewing probe. Biomedical Optics Express.

[23] Muanza TM (2005). Evaluation of radiation-induced oral mucositis by optical coherence tomography. Clinical Cancer Research.

[24] Wilder-Smith P (2007). *In vivo* imaging of oral mucositis in an animal model using optical coherence tomography and optical Doppler tomography. Clinical Cancer Research.

[25] Davoudi B (2012). Noninvasive *in vivo* structural and vascular imaging of human oral tissues with spectral domain optical coherence tomography. Biomedical Optics Express.

[26] Davoudi B (2013). Optical coherence tomography platform for microvascular imaging and quantification: initial experience in late oral radiation toxicity patients. Journal of Biomedical Optics.

[27] Sonis ST (2004). A Biological Approach to Mucositis. The Journal of Supportive Oncology.

[28] Matveev LA (2015). Hybrid M-mode-like OCT imaging of three-dimensional microvasculature *in vivo* using reference-free processing of complex valued B-scans. Optics Letters.

[29] Drogoszewska B, Chomik P, Michcik A, Polcyn A (2013). A standard picture of healthy oral mucosae by direct oral microscopy. Advances in Dermatology and Allergology.

[30] Lucchese A (2015). Fractal analysis of mucosal microvascular patterns in oral lichen planus: a preliminary study. Oral Surgery, Oral Medicine, Oral Pathology, Oral Radiology, and Endodontics.

[31] Scardina GA, Carini F, Noto F, Messina P (2013). Microcirculation in the healing of surgical wounds in the oral cavity. International Journal Oral Maxillofacial Surgery.

[32] Cerny V (2012). Sublingual microcirculation. Applied Cardiopulmonary Pathophysiology.

[33] Abdo IS, Turek Z, Parizkova R, Cerny V (2011). Imaging of the sublingual microcirculation in elderly patients - a pilot study. Applied Cardiopulmonary Pathophysiology.

[34] Milstein DM (2010). Use of sidestream dark-field (SDF) imaging for assessing the effects of high-dose melphalan and autologous stem cell transplantation on oral mucosal microcirculation in myeloma patients. Oral Surgery, Oral Medicine, Oral Pathology, Oral Radiology, and Endodontics.

[35] Gono K (2015). Narrow band imaging: technology basis and research and development history. Clinical Endoscopy.

[36] Kurihara Y (2010). Narrow band imaging of oral mucosa, cancer and pre-cancerous lesions. Dental Medicine Research.

[37] Ni XG, Wang GQ (2016). The role of narrow band imaging in head and neck cancers. Current Oncology Reports.

[38] Takano JH (2010). Detecting early oral cancer: narrowband imaging system observation of the oral mucosa microvasculature. International Journal Oral Maxillofacial Surgery..

[39] Narayan S (2008). Prospective evaluation to establish a dose response for clinical oral mucositis in patients undergoing head-and-neck conformal radiotherapy. International Journal of Radiation Oncology, Biology Physics.

[40] Rege A, Thakor NV, Pathak AP (2012). Optical imaging of microvascular morphology and perfusion. Current Angiogenesis.

[41] Gelikonov VM, Gelikonov GV, Terpelov DA, Shilyagin PA (2012). Electronic interface systems for goals of spectral domain optical coherence tomography. Instruments and Experimental Techniques.

[42] Gelikonov VM, Gelikonov GV, Shilyagin PA (2009). Linear-wavenumber spectrometer for high-speed spectral-domain optical coherence tomography. Optics and Spectroscopy.

[43] Gelikonov VM, Gelikonov GV, Shilyagin PA (2008). Optimization of Fizeau-based optical coherence tomography with a reference Michelson interferometer. Bulletin of the Russian Academy of Sciences: Physics..

[44] Moiseev, A. A. *et al*. Real time OCT-based angiography device with hand-held probe (Conference Presentation). *SPIE Bios. International Society for Optics and Photonics*. 1005315–1005315-1 (2017).

[45] Jia Y (2014). Quantitative optical coherence tomography angiography of choroidal neovascularization in age-related macular degeneration. Ophthalmology.

[46] Conroy L, DaCosta RS, Vitkin IA (2012). Quantifying tissue microvasculature with speckle variance optical coherence tomography. Optics Letters.

